# Unlocking the Functional Potential of Pecan Nut Cake: A Study on Bioactive Peptide Production

**DOI:** 10.3390/foods15020323

**Published:** 2026-01-15

**Authors:** Tianjing Long, Yingjie Xu, Ziang Li, Weimei Kong, Yibo Zhu, Mingxuan Tao, Haibo Luo, Li Cui, Mingjun Sun, Zhen Wu, Xiaoqun Zeng, Daodong Pan, Yuxing Guo

**Affiliations:** 1 Department of Food Science and Technology, School of Food Science and Pharmaceutical Engineering, Nanjing Normal University, Nanjing 210097, China; longtianjing2001@163.com (T.L.); 222712004@njnu.edu.cn (Y.X.); 13782565162@163.com (Z.L.); kongweimei2023@163.com (W.K.); 45017@njnu.edu.cn (M.T.); luohaibo_1216@126.com (H.L.); 2 School of Biological and Food Engineering, Suzhou Institute of Technology, Suzhou 215500, China; centuryrain@126.com; 3 Institute of Agro-Product Processing, Jiangsu Academy of Agricultural Sciences, Nanjing 210014, China; clisu1@163.com; 4 Nanjing You Neng Biotechnology Co., Ltd., Nanjing 211200, China; yangsun0408@outlook.com; 5 School of Food Science and Engineering, Ningbo University, Ningbo 315800, China; wuzhen@nbu.edu.cn (Z.W.); zengxiaoqun@nbu.edu.cn (X.Z.); daodongpan@163.com (D.P.)

**Keywords:** pecan nut cake, lactic acid bacteria fermentation, bioactive peptides, molecular docking

## Abstract

This study examined whether co-fermentation with *Lactobacillus casei* CGMCC 15956 and *Lactobacillus delbrueckii* CGMCC 21287 could enhance the bioactivity of peptides derived from pecan nut cake (PNC) and clarify the underlying mechanisms. The fermented hydrolysate (PNCH) was compared with an unfermented control. PNCH showed higher antioxidant and α-glucosidase inhibitory activities. Total antioxidant capacity increased from 3.17 to 4.81 mM Trolox, and DPPH radical scavenging activity increased from 62.69% to 84.12%. In addition, the IC_50_ value for α-glucosidase inhibition decreased from 7.549 to 4.509 mg/mL. In a mouse model of acute alcohol-induced liver injury, PNCH significantly alleviated liver damage through the synergistic enhancement of antioxidant and α-glucosidase inhibitory activities. Peptidomic analysis identified two representative bioactive peptides, FAGDDAPR (from actin) and LAGNPDDEFRPQ (from cupin domain–containing protein 1), both of which exhibited antioxidant and α-glucosidase inhibitory activities. Additionally, these peptides alleviated H_2_O_2_-induced oxidative stress in Caco-2 cells, significantly improving GSH and MDA levels, as well as SOD activity. Molecular docking suggested potential interactions of these peptides with superoxide dismutase, Keap1, and α-glucosidase. These findings support the high-value utilization of PNC and the development of functional peptide-based ingredients.

## 1. Introduction

Agricultural by-product proteins are abundant yet underexploited as alternative protein sources [1]. Upon hydrolysis, they can release bioactive peptides with antioxidant, antihypertensive, and anti-inflammatory activities, suggesting potential roles in health modulation [2,3]. In recent years, bioactive peptides derived from nut proteins have attracted increasing research interest. Evidence indicates that nut proteins serve as valuable precursors for antioxidant and α-glucosidase inhibitory peptides, among others, and walnut protein peptides have demonstrated their applicability as functional food ingredients [4,5]. In contrast, protein resources in pecan nut (*Carya illinoinensis*) processing by-products remain largely unutilized. Pecan nut cake (PNC), the primary residue of oil extraction, has limited application due to poor solubility and undesirable sensory attributes [6]. Previous studies indicate that PNC is rich in high-quality protein, with a balanced amino acid composition, and contains antioxidant enzyme activities such as superoxide dismutase and catalase, demonstrating its potential to release functional peptides, thereby providing a foundation for the development of functional foods [7]. However, systematic research on peptide production strategies, peptide profiles, and structure–activity relationships is still lacking.

Fermentation, as a green and efficient biotransformation approach, can promote protein degradation and diversify peptide composition, thereby enhancing biological activity [8]. Fermentation of walnut protein by *Bacillus species* has yielded peptides with antioxidative and ACE inhibitory activities, highlighting fermentation as an effective strategy for generating nut-derived bioactive peptides [9]. Lactic acid bacteria (LAB), owing to their safety and well-developed proteolytic systems, offer clear advantages in food fermentation [10]. The coordinated action of intracellular and extracellular proteases in LAB plays a key role in shaping peptide profiles and associated functional properties [11]. LAB fermentation of soy, corn, and pistachio matrices has produced peptides with antioxidant and anti-fatigue activities and has also been shown to reduce protein allergenicity [12]. Nevertheless, the application of LAB to nut by-product proteins for bioactive peptide production remains limited.

We hypothesize that co-fermentation of *L. casei* CGMCC 15956 and *L. delbrueckii* CGMCC 21287 with PNC can enhance the release of bioactive peptides, thereby boosting their antioxidant and α-glucosidase inhibitory activities. Furthermore, PNCH may alleviate liver damage in a murine model of acute alcoholic liver injury. Molecular docking analysis was conducted to explore the potential interactions between PNC-derived bioactive peptides and key proteins such as SOD, Keap1, and α-glucosidase, revealing possible molecular mechanisms. This study aims to optimize the high-value utilization of PNC proteins by employing fermentation technology to enhance their bioactivity, evaluating their potential as functional food ingredients with antioxidant, anti-diabetic, and hepatoprotective effects. To this end, two bioactive peptides were identified from PNC, FAGDDAPR and LAGNPDDEFRPQ, both showing significant activity in antioxidant and α-glucosidase inhibition. These findings further support the development of PNC as a functional food ingredient, providing a theoretical basis for creating high-nutritional-value functional foods.

## 2. Materials and Methods

### 2.1. Materials and Chemicals

PNC (protein:30.12 ± 0.15 g/100 g, fat:20.15 ± 0.17 g/100 g, ash:5.27 ± 0.19 g/100 g, total polyphenols:19.51 ± 2.35 mg/g, and peptides:35.38 ± 1.32 mg/g, Cold pressing) was provided by the Jiangsu Academy of Agricultural Sciences (Nanjing, China). The SDS-PAGE gel preparation kit was purchased from BBI Life Sciences Co., Ltd. (Nanjing, China). Various commercial assay kits, including those for alcohol dehydrogenase (ADH), aldehyde dehydrogenase (ALDH), triglycerides (TG), alanine aminotransferase (ALT), aspartate aminotransferase (AST), superoxide dismutase (SOD), malondialdehyde (MDA), GSH, and for measuring MDA content and SOD activity, were obtained from Nanjing Jiancheng Bioengineering Institute (Nanjing, China). Fifty healthy C57BL/6 mice (3 weeks old, 16–22 g; Animal License No. SCXK (Zhejiang) 2024-0001) were purchased from Zhejiang Viton Lihua Laboratory Animal Technology Co., Ltd. (Jiaxing, China). The peptides with over 95% purity were synthesized by Jei Tai Biotechnology Co., Ltd. (Nanjing, China). The Caco-2 cell line was sourced from the Chinese Academy of Sciences Shanghai Cell Bank (Shanghai, China). All other solvents and chemical reagents were obtained from Sinopharm Chemical Reagent Co., Ltd. (Shanghai, China) and were of analytical grade.

### 2.2. Study of PNC Proteins and Derived Hydrolysates

#### 2.2.1. Analysis of Protein and Amino Acid Composition in PNC

Proteins from PNC were extracted following the procedure of Wu et al. [13]. The proteins were precipitated with trichloroacetic acid and β-mercaptoethanol, then washed with acetone containing protease inhibitors before being lyophilized for further examination. Afterward, the proteins were dissolved in loading buffer and subjected to SDS-PAGE with 5% stacking and 10% separating gels. The gels were stained with Coomassie Brilliant Blue R-250, and protein bands were visualized using a gel documentation system. The amino acid composition of the PNC powder was determined by LC-MS after solvent extraction and derivatization.

#### 2.2.2. Preparation of PNCH by Lactic Acid Bacteria Fermentation

LAB were cultured in a medium containing 5 g PNC, 0.5 g glucose, 0.25 g peptone, and 0.075 g ammonium chloride, prepared at a solid-to-liquid ratio of 1:1.25 (*w*/*v*). The cultures were inoculated with 15% (*v*/*v*) seed culture and incubated at 37 °C for 12 h. The resulting fermentation products were extracted with distilled water at a solid-to-liquid ratio of 1:9 (*w*/*v*) under magnetic stirring at room temperature for 4 h. The mixture was then centrifuged at approximately 3000× *g* for 10 min, and the supernatant was filtered through a 0.22 μm membrane. The filtrate was subsequently freeze-dried for further analyses. The peptide content of the freeze-dried filtrate was determined using the bicinchoninic acid (BCA) assay and expressed as mg/g, while the free amino acid (FAA) content was quantified by the ninhydrin colorimetric method and expressed as μmol/g. Based on these results, individual strains and their combinations were evaluated for hydrolysis efficiency. Finally, the selected combination of *L. casei* CGMCC 15956 and *L. delbrueckii* CGMCC 21287 was co-fermented at a 1:1 (*v*/*v*) ratio, and the resulting hydrolysate was freeze-dried to obtain PNCH for subsequent experiments.

### 2.3. Determination of PNCH Bioactivity

#### 2.3.1. Determination of Chemical Antioxidant Activity In Vitro

Solutions of PNC and PNCH were prepared at different concentration gradients and evaluated for total antioxidant capacity (T-AOC) according to the instructions of the commercial assay kit. In addition, the DPPH radical-scavenging activity of PNC and PNCH was determined following the method of Gang et al. [14] with slight modifications. Briefly, 1 mL of 0.2 mM DPPH solution was mixed with the sample solution and incubated in the dark at 25 °C for 30 min. The absorbance was measured at 517 nm, where A_i_, A_j_, and A_0_ represented the absorbance of the sample, the sample replaced with distilled water, and the DPPH solution alone, respectively. The radical scavenging activity was calculated according to Equation (1).

(1) $$DPPH\;Radical\;Scavenging\;Activity\left(\%\right)=\left[1-\frac{\left(A_{i}-A_{j}\right)}{A_{0}}\right]\times 100\%$$

#### 2.3.2. Determination of α-Glucosidase Inhibition Activity In Vitro

The α-glucosidase inhibitory activity of PNC and PNCH was determined as previously described with slight modifications [15]. Briefly, in a 96-well plate, 20 μL of phosphate buffer (0.1 mol/L, pH 6.8), 20 μL of α-glucosidase solution (0.2 U/mL), and the sample solution were mixed and incubated at 37 °C for 5 min. Subsequently, 20 μL of PNPG (5 mg/mL) was added and the mixture was further incubated for 30 min. The reaction was terminated by adding 80 μL of Na_2_CO_3_ (0.2 mol/L), and absorbance was measured at 405 nm. OD_blank_, OD_negative_, OD_background_, and OD_sample_ represented the blank control (PBS + Na_2_CO_3_), negative control (buffer instead of sample), background control (buffer instead of enzyme), and sample group, respectively. The inhibition rate was calculated according to Equation (2).

(2) $$\alpha -Glucosidase\;Inhibitory\;Activity\left(\%\right)=\left(1-\frac{{OD}_{Sample}-{OD}_{Background}}{{0D}_{Negative}-{OD}_{Blank}}\right)\times 100\%$$

#### 2.3.3. Animal Experimental Design

Fifty healthy ICR mice were maintained under controlled conditions (20–26 °C, 40–70% relative humidity, 12 h light/dark cycle) and acclimated for 7 days prior to experimentation. The animals were randomly divided into five groups (*n* = 10): Control, Model, silymarin (50 mg/kg, SIL), high-dose PNCH (750 mg/kg, PNCH-H), and low-dose PNCH (150 mg/kg, PNCH-L). All groups received their respective treatments by oral gavage once daily for 7 consecutive days. On the seventh day, 2 h after the final administration, all groups except the Control group were given ethanol (53% *v*/*v*, Beijing Red Star Erguotou liquor) at a dose of 20 mL/kg body weight by gavage, followed 6 h later by an additional ethanol dose of 10 mL/kg to induce acute alcoholic liver injury. After a 16 h fasting period, the mice were anesthetized, and blood and liver samples were collected.

#### 2.3.4. Serum and Liver Biochemical Index

Mouse serum was obtained by centrifugation at 3000× *g* for 10 min at 4 °C. The levels of ALT and AST in serum and those of ADH, ALDH, SOD, MDA, TG, and protein in liver homogenates were determined using commercial assay kits.

#### 2.3.5. Histological Analysis

Liver tissues were fixed with 4% paraformaldehyde, dehydrated, and sectioned using paraffin embedding. Hematoxylin and eosin (H&E) staining.

### 2.4. Purification and Activity Identification

#### 2.4.1. Identification of Peptide Sequences from PNCH

PNCH, the crude hydrolysate prepared as described in Section 2.2.2, was processed by reducing, alkylating, and desalting using C18 cartridges, followed by quantification prior to mass spectrometric analysis. The processed samples were analyzed by nano LC-HRMS on a C18 column, employing a 4–95% acetonitrile gradient over 66 min at a flow rate of 600 nL/min. Mass spectra were acquired over an *m/z* range of 100–1500, with MS1 and MS2 resolutions set to 120,000 and 15,000, respectively.

#### 2.4.2. Peptide Sequence Analysis and Activity Prediction

The bioactivity of peptides identified from the PNCH was predicted using PeptideRanker (http://distilldeep.ucd.ie/PeptideRanker/, accessed on 15 November 2025), where scores approaching 1 indicate a higher likelihood of biological activity. Peptide segments with PeptideRanker scores exceeding 0.95 were selected for further analysis. BIOPEP-UWM (https://biochemia.uwm.edu.pl/biopep-uwm/, accessed on 15 November 2025), encompassing all reported bioactive peptides, was utilized to ascertain the novelty of the obtained peptides. The physicochemical properties of the peptides were predicted using ProParam (http://web.expasy.org/protparam/, accessed on 15 November 2025). ToxinPred (https://webs.iiitd.edu.in/raghava/toxinpred3/, accessed on 15 November 2025) was employed to predict peptide toxicity based on Swiss-Prot data. AlgPred (https://webs.iiitd.edu.in/raghava/algpred2/, accessed on 15 November 2025) was utilized to evaluate the allergenicity of the peptides based on their amino acid sequences.

#### 2.4.3. Peptide Synthesis and Characterization

Based on molecular docking scores, two peptides (purity > 95%) were synthesized by Nanjing Jiepeptide Biotechnology Co., Ltd. (Nanjing, China) using solid-phase peptide synthesis, and their total antioxidant capacity, DPPH radical scavenging activity, and α-glucosidase inhibitory activity were subsequently evaluated.

#### 2.4.4. Protective Effects of Peptides on Oxidative Stress in Caco-2 Cells

Caco-2 cells were cultured in DMEM supplemented with fetal bovine serum (FBS), penicillin–streptomycin, and non-essential amino acids (NEAA) (20:1:1:80, *v*/*v*) at 37 °C in a 5% CO_2_ incubator. Cells were seeded in 6-well plates at 1 × 10^5^ cells/mL (2 mL per well) and cultured for 24 h. To establish an oxidative stress model, cells were treated with 200 μM H_2_O_2_ for 2 h. After treatment, the medium was replaced with fresh culture medium and cells were incubated for an additional 24 h. For peptide intervention, cells were incubated with fresh medium containing 400 μg/mL different peptides following H_2_O_2_ exposure. Additionally, for the vitamin C (VC) group, cells were incubated with fresh medium containing 400 μg/mL VC following H_2_O_2_ exposure. Cells in the control group received PBS instead of H_2_O_2_ and peptides. Cell viability was determined using the MTT assay. After incubation, cells were collected, lysed, and intracellular GSH and MDA contents together with SOD activity were quantified using assay kits.

#### 2.4.5. In Silico Interaction Analysis of Peptides and Target Proteins

Molecular docking was conducted to explore the interactions between selected peptides and target proteins, including SOD (PDB ID:2ADQ), the Keap1-Nrf2 complex (PDB ID:8IXS), and α-glucosidase (PDB ID:3WY1). The peptide structures were built and energy-minimized in ChemBio3D Ultra 20.0 (PerkinElmer Inc., Waltham, MA, USA) and subsequently parameterized in AutoDockTools 1.5.6 (Scripps Research Institute, La Jolla, CA, USA). The crystal structures of the targets were obtained from the RCSB Protein Data Bank and preprocessed in PyMOL 1.7 by removing water molecules and ligands, adding hydrogens, and optimizing geometry. Gasteiger charges and AMBER atom types were assigned, and the receptor files were saved in PDBQT format.

Docking simulations were performed in AutoDock Vina 1.1.2 using semi-flexible docking. The grid centers for SOD, Keap1-Nrf2, and α-glucosidase were set at (−8.777, 36.844, 215.925), (23.089, 61.601, 38.392), and (−6.693, −17.460, 19.975), respectively, with grid boxes covering the entire binding pockets. Binding affinities were evaluated using Vina’s scoring function, and the lowest-energy conformations were selected for further analysis. The resulting complexes were visualized and analyzed in PyMOL v2.5.2 to identify key binding interactions.

### 2.5. Statistical Analysis

All experimental data are expressed as mean ± standard deviation (SD). Graphical representations were prepared using OriginPro 2021 (OriginLab, Northampton, MA, USA) and GraphPad Prism 8.0 (GraphPad Software, Boston, MA, USA). Statistical analyses were conducted using one-way analysis of variance (ANOVA) followed by Dunnett’s multiple comparison test in SPSS 26.0 (IBM Corp., Armonk, NY, USA). All experiments were performed in triplicate, and statistical significance was defined as *p* < 0.05.

## 3. Results

### 3.1. Protein and Amino Acid Composition of PNC

**Figure 1:**
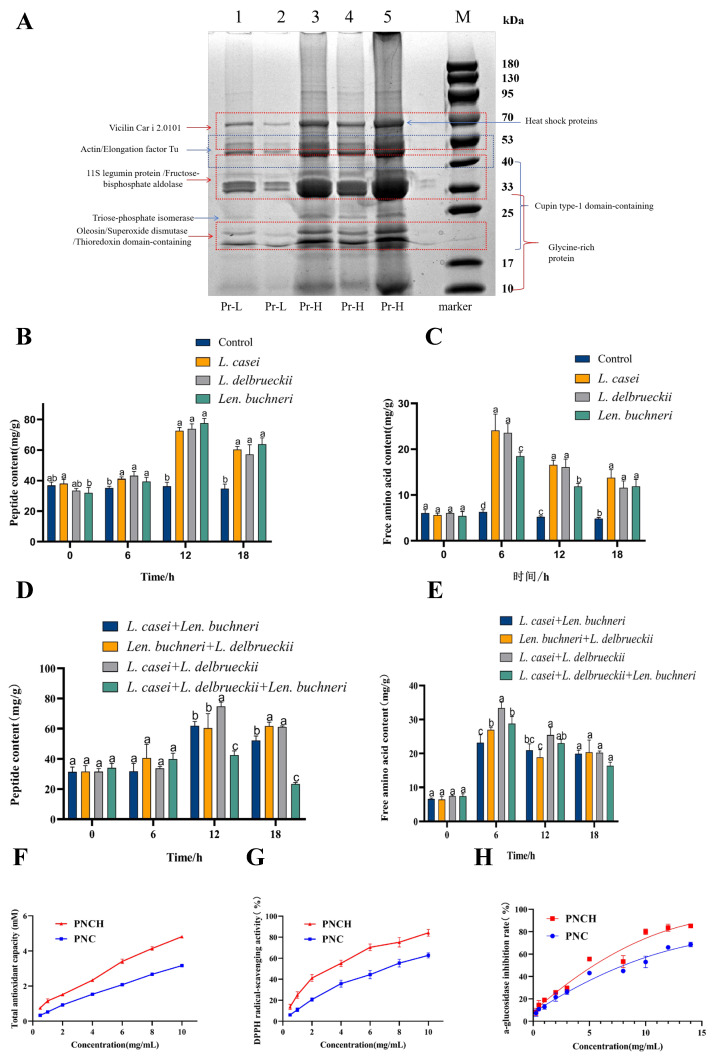
(**A**) SDS-PAGE profile of PNC proteins. Lane M: molecular weight marker; Lanes 1–2: pecan peptides at a concentration of 0.05 g/mL; Lanes 3–5: pecan peptides at a concentration of 0.1 g/M (**A**); (**B**–**E**) effects of monoculture and co-culture fermentations with different bacterial strains on peptide and free amino acid contents in PNC. Different letters above the bars indicate significant differences (*p* < 0.05) according to Duncan’s multiple range test; (**F**–**H**) antioxidant capacity and α-glucosidase inhibitory activity of bifunctional peptides from PNC.

PNC exhibits a high crude protein content (30.12 ± 0.15 g/100 g), making protein one of its primary nutritional components. SDS-PAGE analysis revealed that pecan meal proteins displayed a broad molecular weight distribution ranging from 10 to 70 kDa (Figure 1A). The most intense bands were observed in the 50–70 kDa region, likely corresponding to 7S vicilin storage proteins and HSP70. Prominent bands in the 30–40 kDa range may represent 11S legumin subunits, fructose-bisphosphate aldolase, and cupin superfamily proteins, while those in the 40–50 kDa region were attributed to actin and elongation factor Tu. Proteins of 25–30 kDa were consistent with triose-phosphate isomerase, and those in the 20–30 kDa range likely included cupins and low-molecular-weight metabolic enzymes. Multiple bands within 10–30 kDa were associated with glycine-rich proteins and oleosins, typically related to oil body structures. Collectively, these findings indicate that pecan meal proteins are predominantly storage proteins, accompanied by structural and metabolic proteins. PNC is rich in essential amino acids, with particularly high levels of Glu, Thr, and Cys (Table 1). Amino acids such as Gly, Ser, Glu, Tyr, and Arg have been implicated in α-glucosidase inhibition [16], whereas Pro, Glu, Cys, Leu, and Thr are known to contribute to antioxidant activity [17]. The results indicate that PNC is a promising precursor of bioactive peptides with both antioxidant and α-glucosidase inhibitory potential.

### 3.2. Strain-Specific and Synergistic Effects on Peptide and Amino Acid Production

During PNC fermentation, peptide content exhibited a time-dependent increase, reaching its maximum at 12 h for all strains (Figure 1B–E). The total peptide contents of *L. casei* CGMCC 15956, *L. delbrueckii* CGMCC 21287, and *L. buchneri* CGMCC NO.21286 (72.59 ± 2.07, 73.93 ± 3.20, and 77.59 ± 2.98 mg/g, respectively) were not significantly different from that of the *L. casei*–*L. delbrueckii* co-culture (74.75 ± 2.79 mg/g, *p* > 0.05), indicating a comparable extent of overall protein hydrolysis at the endpoint. However, co-culture of *L. casei* CGMCC 15956 and *L. delbrueckii* CGMCC 21287 produced markedly higher FAA concentrations at both 6 h (33.34 ± 1.75 mg/g) and 12 h (25.42 ± 2.20 mg/g) compared with single-strain or other mixed fermentations (*p* < 0.05). This enhanced FAA accumulation, despite similar total peptide yields, indicates a more efficient and comprehensive proteolytic process, reflecting synergistic peptidase activity between the two strains that promotes deeper protein degradation.

### 3.3. Antioxidant Capacity and α-Glucosidase Inhibitory Activity of PNCH

Fermentation significantly enhanced the antioxidant activity of PNCH. At 10 mg/mL, T-AOC increased from 3.17 ± 0.08 mM Trolox (unfermented) to 4.81 ± 0.06 mM (fermented), and the DPPH radical scavenging rate increased from 62.69 ± 2.40% to 84.12 ± 3.22%. Additionally, the α-glucosidase inhibitory activity of PNCH improved significantly, with the IC50 value decreasing from 7.549 mg/mL to 4.509 mg/mL, indicating enhanced potential for antidiabetic effects.

### 3.4. Evaluation of PNCH to Alleviate Acute Alcoholic Liver Injury in Mice

**Figure 2:**
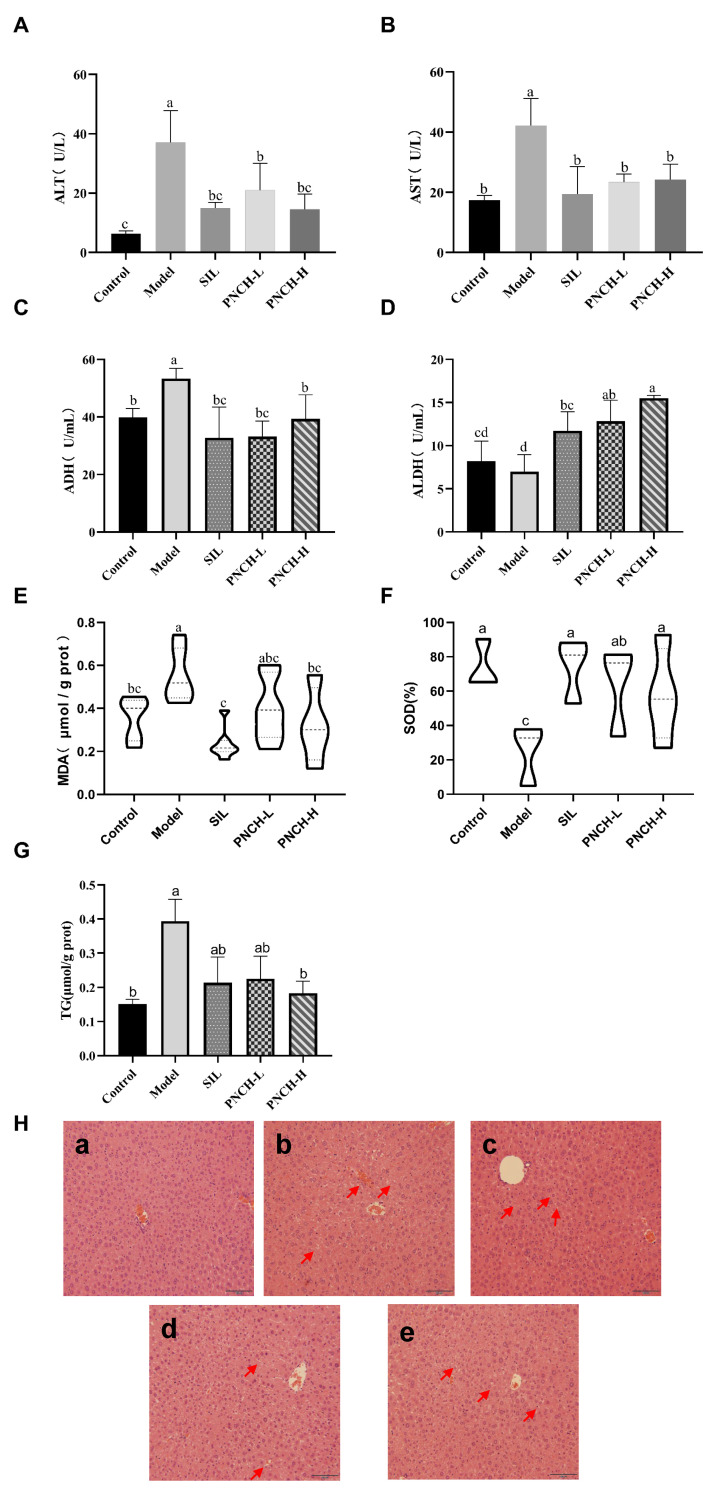
Effect of PNCH on alcohol metabolism, liver functional markers, hepatic oxidative stress, and histopathological changes in mice. (**A**) Plasma ALT level; (**B**) Plasma AST level; (**C**) Hepatic ADH activity; (**D**) Hepatic ALDH activity; (**E**) Hepatic MDA content; (**F**) Hepatic SOD activity; (**G**) Hepatic TG content; (**H**) Liver histopathology (H&E staining): (**a**) Control group; (**b**) Model group; (**c**) SIL group; (**d**) PNCH-L group; (**e**) PNCH-H group. Different letters (a–d) on the top of columns indicate a significant difference at *p* < 0.05.

Throughout the experiment, all mice in groups exhibited normal behavior with no signs of toxicity or mortality, indicating good tolerance to PNCH. Ethanol exposure led to slight body weight loss and a significant increase in liver index, confirming the successful induction of acute alcoholic liver injury (Figure S1). Ethanol exposure markedly elevated serum ALT (37.08 ± 10.76 U/L) and AST (42.12 ± 9.02 U/L) levels compared with the control group (6.39 ± 0.89 and 17.37 ± 1.60 U/L, respectively; *p* < 0.05), indicating substantial hepatocellular injury (Figure 2A,B). Administration of PNCH effectively reduced ALT and AST activities by approximately 50%, demonstrating a pronounced hepatoprotective effect. Consistently, hepatic triglyceride (TG) content was significantly increased in the alcohol-treated group (0.50 μmol/g protein) relative to the control (0.30 μmol/g protein), whereas PNCH supplementation dose-dependently alleviated lipid accumulation (Figure 2G). Moreover, PNCH treatment enhanced ALDH activity (15.50 ± 0.34 U/mL in the PNCH-H) and restored hepatic antioxidant capacity, as evidenced by the reduction in MDA levels (from 0.55 ± 0.12 to 0.36 ± 0.10 μmol/g protein) and the recovery of SOD activity (from 25.19 ± 17.75% to 73.70 ± 14.34%) (Figure 2C–F). Histopathological observations (Figure 2H) further confirmed that PNCH markedly attenuated ethanol-induced hepatocyte swelling, cytoplasmic vacuolation, and lipid droplet accumulation, particularly in the high-dose group, which displayed nearly normal hepatic architecture. Collectively, these findings demonstrate that PNCH effectively mitigates acute alcohol-induced liver injury by modulating oxidative stress, lipid metabolism, and ethanol detoxification pathways.

### 3.5. Peptide Sequence Identification and Its Activity Prediction

**Figure 3:**
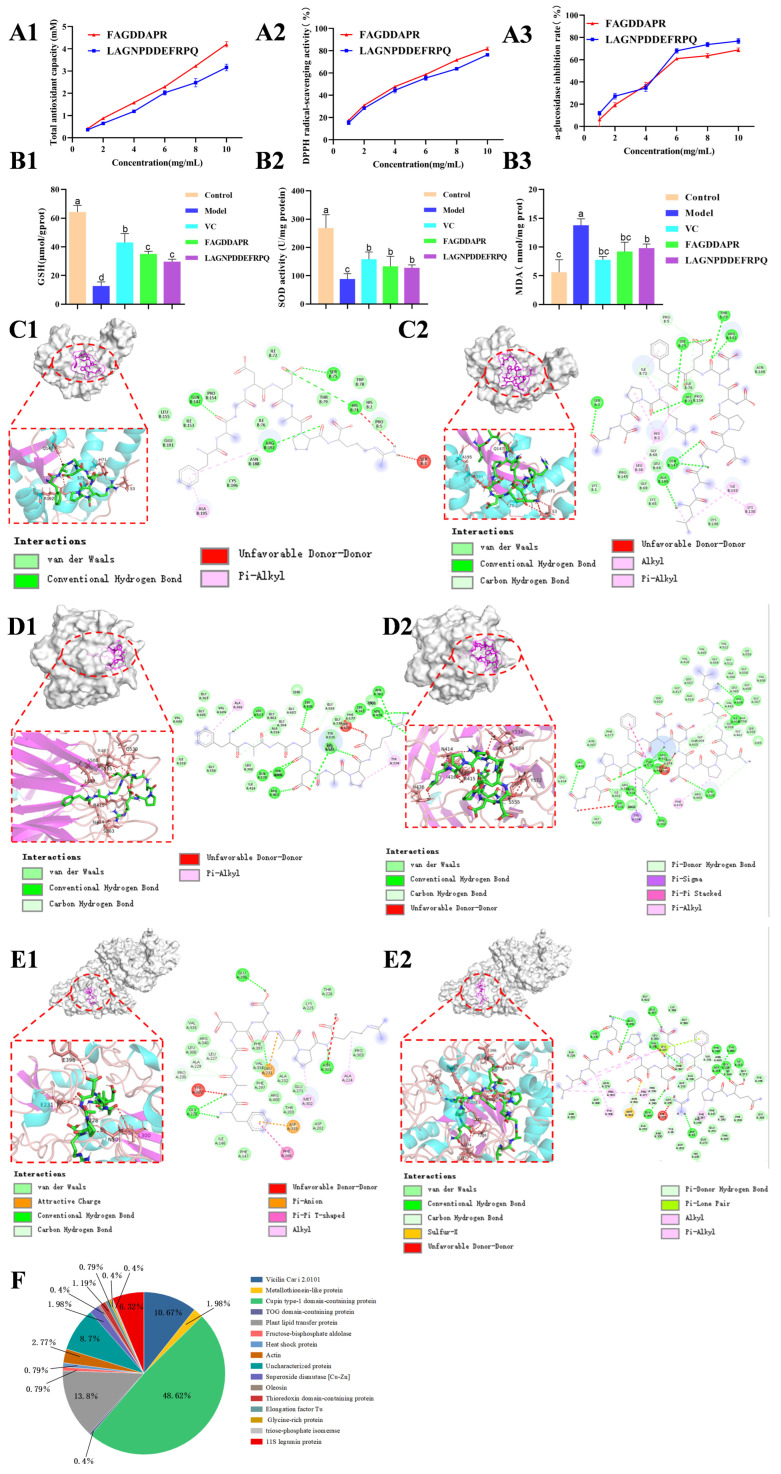
In vitro antioxidant and α-glucosidase inhibitory activities of FAGDDAPR and LAGNPDDEFRPQ, their protective effects against oxidative stress in H_2_O_2_-induced Caco-2 cells, and molecular docking analysis. In vitro activities: (**A1**) Total antioxidant capacity; (**A2**) DPPH radical scavenging activity; (**A3**) α-glucosidase inhibitory activity; In H_2_O_2_-induced Caco-2 cells: (**B1**) intracellular GSH levels; (**B2**) SOD activity; (**B3**) MDA content; (**C1**,**C2**) Molecular docking diagrams of the peptides with SOD: (**C1**) FAGDDAPR and (**C2**) LAGNPDDEFRPQ; (**D1**,**D2**) Docking conformations of the antioxidant peptides within the Keap1 protein: (**D1**) FAGDDAPR and (**D2**) LAGNPDDEFRPQ; (**E1**,**E2**) Molecular docking diagrams of the peptides with α-glucosidase: (**E1**) FAGDDAPR and (**E2**) LAGNPDDEFRPQ; (**F**) Precursor protein analysis of peptides identified in PNC. Different letters (a–d) on the top of columns indicate a significant difference at *p* < 0.05.

By LC-MS/MS, 253 peptides were identified from PNCH (Figure S2A), primarily consisting of 7–14 amino acids and less than 1.5 kDa (Figure S2B,C). These peptides mainly originated from Cupin type-1 domain proteins (48.6%), plant lipid transfer proteins (13.8%), and vicilin Car i 2.0101 (10.7%) (Figure 3F). Some peptides are associated with allergenic potential. Fermentation degraded these proteins, reducing allergenicity and enhancing bioactive peptide formation. PNC is a promising, safer peptide source for functional foods and nutraceuticals.

Peptide bioactivity is closely associated with structural characteristics such as molecular weight, amino acid composition, and terminal residues. the identified peptides were particularly enriched in Glu, Gly, and Arg. Among these residues, Glu and Arg are known to enhance antioxidant activity through radical scavenging and metal ion chelation mechanisms [18], whereas Gly may facilitate enzyme–substrate interactions and frequently occurs in DPP-IV inhibitory peptides [19] (Figure S2D–F). In addition, N-terminal hydrophobic residues (Leu, Val, Ile) are reported to improve membrane permeability and enzyme affinity [20], while C-terminal Glu and Arg may stabilize peptide–protein interactions [21].Collectively, these compositional and structural characteristics provide a molecular basis for the potential bioactivities of the identified peptides.

All 253 identified peptide sequences were subjected to PeptideRanker analysis, and a considerable number scored above 0.5, indicating high potential bioactivity. After toxicity and allergenicity screening, four peptides were identified as promising bioactive candidates (Table S1). Among them, FAGDDAPR has previously been reported to exhibit ACE-inhibitory and alcohol dehydrogenase-activating activities, while the remaining three peptides, though not previously characterized, displayed favorable structural features and high predictive scores, suggesting strong application potential. The physicochemical characteristics of these peptides are summarized in Table S1. Considering that short-chain peptides typically demonstrate enhanced absorption and in vivo stability [22], FAGDDAPR (8 amino acids, derived from actin) and LAGNPDDEFRPQ (12 amino acids, derived from cupin domain-containing protein 1) were subsequently selected for molecular docking analysis to further elucidate their binding mechanisms and potential biological targets.

### 3.6. The Activity Validation of Peptides

LAGNPDDEFRPQ and FAGDDAPR were chemically synthesized, and their bioactivities were systematically evaluated (Figure 3(A1–A3)). At a concentration of 10 mg/mL, T-AOC of LAGNPDDEFRPQ and FAGDDAPR reached 3.16 ± 0.15 mM Trolox and 4.20 ± 0.12 mM Trolox, respectively. Both peptides also exhibited pronounced DPPH radical scavenging activity, with IC_50_ values of 6.756 mg/mL and 7.447 mg/mL, respectively. Furthermore, they demonstrated significant α-glucosidase inhibitory activity, with IC_50_ values of 4.009 mg/mL and 4.650 mg/mL, respectively. These results indicate that the synthesized peptides possess dual bioactivities, acting as both antioxidants and α-glucosidase inhibitors, thereby highlighting their potential as natural functional ingredients.

As shown in Figure S4, MTT assays demonstrated acceptable cytotoxicity at 400 μg/mL peptide concentration, and 200 μM H_2_O_2_, which induced a moderate but significant reduction in cell viability, was therefore selected as the oxidative stress–inducing condition for subsequent experiments. As shown in Figure 3(B1–B3), H_2_O_2_ challenge significantly disturbed intracellular redox homeostasis in Caco-2 cells, as indicated by a marked reduction in GSH content and SOD activity, along with a pronounced increase in MDA levels. Specifically, GSH levels decreased from 64.24 ± 4.65 to 12.71 ± 2.82 μmol/g prot, while SOD activity declined by approximately 67.1% following H_2_O_2_ exposure. In contrast, MDA content increased from 5.62 ± 2.16 to 13.77 ± 1.12 nmol/mg prot. Pretreatment with FAGDDAPR or LAGNPDDEFRPQ significantly counteracted H_2_O_2_-induced oxidative damage. Both peptides restored GSH levels to 35.03 ± 1.84 and 29.62 ± 1.78 μmol/g prot, respectively. Notably, SOD activity was significantly enhanced, increasing by 50.9% in the FAGDDAPR-treated group and 44.6% in the LAGNPDDEFRPQ-treated group relative to the H_2_O_2_-damaged group (*p* < 0.05). Meanwhile, MDA accumulation was concomitantly reduced to 9.22 ± 1.61 and 9.79 ± 0.72 nmol/mg prot, respectively. These results indicate that FAGDDAPR and LAGNPDDEFRPQ exert cytoprotective effects by reinforcing antioxidant defenses and alleviating lipid peroxidation in oxidatively stressed Caco-2 cells.

### 3.7. Molecular Docking of Peptides with SOD

FAGDDAPR and LAGNPDDEFRPQ exhibited binding energies below −5.0 kcal/mol when docked with SOD, indicating thermodynamically favorable interactions (Table 2). Specifically, FAGDDAPR formed five hydrogen bonds with Ser-3, His-71, Ser-75, Gln-147, and Arg-192, together with four hydrophobic contacts involving Pro-5, Ile-76, Glu-191, and Ala-195 (Figure 3(C1); Table 2). LAGNPDDEFRPQ established six hydrogen bonds with Ser-3, His-71, Ser-75, Gln-147, Arg-192, and Ala-195, and engaged in hydrophobic interactions with Leu-38, Ile-72, Ile-76, and Pro-154 (Figure 3(C2); Table 2). These hydrogen bonding and hydrophobic contacts, as key noncovalent forces, are essential for maintaining the stability of protein-ligand complexes.

### 3.8. Molecular Docking of Peptides with Keap1

Both peptides showed strong binding to Keap1 with binding energies below −7.0 kcal/mol, suggesting stable interactions (Table 2). FAGDDAPR formed hydrogen bonds with Ser-363, Asn-414, Arg-415, Arg-483, Ser-508, Gln-530, Ser-555, and Leu-557, along with hydrophobic interactions with Tyr-334, Ala-366, and Tyr-535 (Figure 3(D1); Table 2). LAGNPDDEFRPQ established hydrogen bonds with Tyr-334, Asn-414, Arg-415, Ile-416, His-436, Ser-555, Tyr-572, and Val-604, and hydrophobic contacts with Tyr-334, Tyr-525, and Phe-577 (Figure 3(D2); Table 2). These interactions contribute to the stability of the peptide-Keap1 complexes and may be linked to their antioxidant activity.

### 3.9. Molecular Docking of Peptides with Alpha-Glucosidase

Docking analysis revealed that both peptides, FAGDDAPR and LAGNPDDEFRPQ, formed stable complexes with α-glucosidase, with binding free energies of −7.9 kcal/mol and −5.8 kcal/mol, respectively (Table 2). FAGDDAPR formed five hydrogen bonds with Gly-228, Glu-231, Leu-300, Asn-301, and Glu-396, and seven hydrophobic interactions with Phe-147, Thr-203, Ala-224, Leu-227, Met-302, Tyr-389, and Phe-397 (Figure 3(E1); Table 2). LAGNPDDEFRPQ established eleven hydrogen bonds with Gly-228, Glu-231, and Glu-396, and twelve hydrophobic contacts with Phe-147, Phe-166, Tyr-235, Tyr-308, and Phe-397 (Figure 3(E2); Table 2). These interactions likely contribute to the stability of the peptide–α-glucosidase complexes, supporting their potential inhibitory effects.

## 4. Discussion

In recent years, proteolytic microbial fermentation has attracted attention for its dual potential to degrade food allergens and release bioactive peptides [23]. To understand how LAB contribute to allergen reduction in fermented foods, systematic characterization of their hydrolysates is necessary. LAB possess endogenous protease systems that cleave IgE-binding epitopes in food proteins, generating peptides with reduced immunogenicity and mitigating allergic responses [11]. For example, *Lactobacillus delbrueckii* subsp. bulgaricus efficiently hydrolyzes milk protein epitopes and is used in hypoallergenic dairy products [24]. However, the use of LAB in plant-derived proteins remains less explored. In this study, MS analysis showed that LAB fermentation primarily produced peptides from PNC proteins containing cupin_1 domains, non-specific lipid transfer proteins (nsLTPs), and 7S vicilin-like proteins (Car i 2.0101), known or predicted major pecan allergens [25]. These findings suggest that LAB proteases preferentially target allergen-associated regions, reducing the allergenic potential of pecan proteins by generating low-molecular-weight peptides with lower immunoreactivity. Similar results have been observed in other plant systems, such as reduced antigenicity of soybean proteins by mixed cultures of *L. casei*, yeast, and *Bacillus subtilis* [26], and hydrolysis of β-conglycinin and glycinin epitopes in soy by *L. plantarum B1*-6 [27] and *L. brevis* [28]. Additionally, *Pediococcus acidilactici* XZ31 decreased IgE reactivity in wheat proteins [29]. Our findings expand the allergen-reducing capability of LAB to pecans, supporting their potential in hypoallergenic nut-based products.

The proteolytic activity of LAB is highly species- and strain-dependent, as differences in protease system composition determine substrate specificity, cleavage site preference, and peptide release profiles [30]. In this study, PNC, which is rich in protein, contains a diverse range of amino acids, including glutamic acid, aspartic acid, threonine, methionine, and cysteine. These amino acids are known to play key roles in antioxidant defense, enzyme inhibition, and various metabolic processes [31]. Based on peptide and free amino acid content, *L. casei* CGMCC 15956 and *L. delbrueckii* CGMCC 21287 were identified as an effective combination for fermentation. *L. casei* has been extensively reported to generate antioxidant and multifunctional peptides; for instance, Shu et al. [32] observed that fermentation of goat milk with *L. casei* L61 increased DPPH radical scavenging activity by more than 60%, while Chandana Kumari et al. [33] demonstrated α-glucosidase inhibitory activity in fermented dough. Meanwhile, *L. delbrueckii* exhibits strong hydrolytic capacity, particularly toward proline-rich substrates [34]. Supporting this, Zhang et al. [35] showed that its cell-envelope proteinases enhanced the antioxidant potential of soybean protein hydrolysates, and Qian et al. [36] reported that 3–5 kDa peptides from *L. delbrueckii*-fermented skim milk displayed notable antioxidant effects. Consistent with these findings, co-fermentation of PNC by *L. casei* CGMCC 15956 and *L. delbrueckii* CGMCC 21287 in the present study significantly improved the antioxidant and α-glucosidase inhibitory properties of the hydrolysates, suggesting that combining LAB strains with complementary proteolytic traits can enhance the functional peptide profiles of plant-derived substrates.

Nutritional intervention has attracted increasing attention as a strategy to mitigate ALD due to its efficacy and low risk of side effects [37,38]. As the primary site of ethanol metabolism, the liver is particularly susceptible to alcohol-induced injury, with excessive intake leading to acute ALD characterized by oxidative stress, redox imbalance, and inflammation [39]. Previous studies have shown that α-glucosidase inhibition can delay glucose absorption, improve insulin sensitivity, and alleviate ethanol-induced metabolic disturbances and hepatic stress [40,41]. In the present ALD mouse model, HE staining revealed pronounced hepatocellular edema and steatosis in the model group, whereas peptide-treated mice exhibited markedly attenuated pathological changes, with hepatic architecture approaching normal. Consistently, biochemical analyses showed that peptide administration at both low and high doses enhanced ALDH activity, reduced serum AST, ALT, and TG levels, increased SOD activity, and decreased hepatic MDA accumulation. Collectively, these results indicate that PNC-derived peptides exert hepatoprotective effects in acute ALD, likely through coordinated antioxidant and metabolic regulatory mechanisms.

Sequence analysis identified two PNC-derived peptides, FAGDDAPR and LAGNPDDEFRPQ, containing residues commonly linked to antioxidant and hypoglycemic activities. FAGDDAPR is a short peptide with an N-terminal composed of phenylalanine, alanine, and glycine, likely promoting radical scavenging and enzyme interactions [42]. The central Asp-Asp dipeptide introduces negative charges, enhancing electrostatic binding, while the C-terminal Pro-Arg motif is associated with α-glucosidase inhibition [43]. In contrast, LAGNPDDEFRPQ has a longer, more complex sequence: its hydrophobic N-terminal residues provide conformational flexibility, central acidic residues offer multiple charge-mediated binding sites, and C-terminal Pro, Arg, and Gln residues may stabilize interactions through hydrogen bonding and hydrophobic contacts [44]. Both peptides contain proline, which enhances structural rigidity and bioactivity. These sequence features provide a basis for molecular docking analysis.

Molecular docking is a widely used method for screening bioactive peptides and understanding their mechanisms by predicting ligand-receptor interactions and visualizing molecular conformations [45]. Negative binding energies indicate spontaneous binding, with values below −5.0 kcal/mol considered favorable, and below −7.0 kcal/mol suggesting strong interactions. Docking results showed that both peptides interacted with SOD, Keap1, and α-glucosidase, with binding free energies below −5.0 kcal/mol for SOD, indicating favorable interactions. Predicted hydrogen bonds with residues His-71, Ser-75, Gln-147, and Arg-192 suggest recurring binding sites within SOD. These interactions imply that FAGDDAPR and LAGNPDDEFRPQ may stabilize SOD’s conformation or influence its catalytic activity, enhancing antioxidant effects.

The Keap1-Nrf2-ARE pathway regulates oxidative stress and inflammation to maintain cellular redox balance [46]. Docking to the Keap1 Kelch domain revealed binding energies below −7.0 kcal/mol for both peptides, indicating favorable interactions. Several residues involved in Nrf2 binding, including Arg-380, Arg-415, Arg-483, Ser-508, Tyr-334, and Tyr-572, were predicted to interact with the peptides [47]. These overlaps suggest that FAGDDAPR and LAGNPDDEFRPQ may compete with Nrf2’s ETGE motif for Keap1 binding, potentially destabilizing the Keap1-Nrf2 complex and promoting Nrf2 release. However, the relevance of this competition under physiological conditions requires further investigation, considering factors like peptide stability, bioavailability, and binding affinity. These results align with previous findings by Liu et al. [48], who reported that dietary peptides modulate oxidative stress via the Keap1-Nrf2 pathway.

Molecular docking indicated that both peptides, FAGDDAPR and LAGNPDDEFRPQ, interact spontaneously with α-glucosidase, forming thermodynamically favorable complexes with binding energies of −7.9 and −5.8 kcal/mol, respectively. The shorter peptide, FAGDDAPR, exhibited higher binding affinity, likely due to its compact structure facilitating efficient accommodation in the substrate-binding groove. It formed five hydrogen bonds and seven hydrophobic interactions with key residues such as Gly-228, Glu-231, Leu-300, Asn-301, and Glu-396, which are important for substrate recognition and catalysis [49]. This suggests that FAGDDAPR may inhibit α-glucosidase by partially blocking substrate access or altering its catalytic conformation. In contrast, LAGNPDDEFRPQ formed a broader interaction network, with eleven hydrogen bonds and twelve hydrophobic contacts with residues like Gly-228, Glu-231, Tyr-235, and Phe-397. Its higher binding energy may indicate greater conformational flexibility and steric hindrance, potentially reducing binding stability and efficiency.

To verify the computational predictions, FAGDDAPR and LAGNPDDEFRPQ were chemically synthesized and evaluated for antioxidant and α-glucosidase inhibitory activities in vitro. Both peptides displayed measurable activities that were comparable to, or in some cases exceeded, those of previously reported bioactive peptides. For instance, their antioxidant capacity was similar to that of MLWQYKPK and VWYA [50], supporting the established contribution of acidic and aromatic residues (Asp, Glu, Tyr, Trp) to radical stabilization [51]. Likewise, their α-glucosidase inhibitory effects were on par with NNNPFKF [52] and WESGPW [53], emphasizing the importance of proline, glycine, and acidic residues in modulating enzyme activity. Together, these findings provide experimental support for the predicted bioactivities and highlight sequence–structure features relevant to functional peptide design.

The antioxidant activity of FAGDDAPR and LAGNPDDEFRPQ was confirmed by their protective effects in an H_2_O_2_-induced Caco-2 cell model. Oxidative stress disrupts cellular redox homeostasis by impairing antioxidant systems and causing lipid peroxidation [54]. The peptides’ attenuation of oxidative damage suggests their antioxidant effects go beyond radical scavenging, potentially involving modulation of intracellular defense systems. Molecular docking revealed interactions with SOD and components of the Keap1-Nrf2 pathway, linking the in vitro and cellular findings. These results highlight the potential of the peptides as functional antioxidants. However, further studies are needed to confirm these effects in vivo.

The shorter peptide FAGDDAPR outperformed LAGNPDDEFRPQ in both docking and experimental assays. Its smaller size and reduced conformational flexibility may facilitate more efficient access to enzyme active sites and stable binding [55]. In contrast, longer peptides may face steric hindrance or conformational fluctuations, reducing their affinity and specificity. These findings align with previous reports that short peptides typically exhibit stronger radical-scavenging and enzyme-inhibitory activities [44]. Overall, FAGDDAPR demonstrated superior antioxidant and hypoglycemic potential, highlighting short, structurally simple peptides as promising candidates for functional ingredient development.

## 5. Conclusions

This study showed that co-fermentation of PNC with *L. casei* CGMCC 15956 and *L. delbrueckii* CGMCC 21287 effectively promoted the generation of bioactive peptides. The PNCH exhibited enhanced antioxidant and α-glucosidase inhibitory activities in vitro and showed hepatoprotective effects in an acute alcohol-induced liver injury mouse model. Peptidomic analysis indicated that LAB fermentation preferentially degraded allergen-related proteins, reducing potential allergenicity. Two peptides, FAGDDAPR and LAGNPDDEFRPQ, were identified and confirmed to possess dual antioxidant and α-glucosidase inhibitory activities in vitro. Molecular docking suggested possible interactions of these peptides with Keap1 and α-glucosidase, providing mechanistic support for their bioactivities. Overall, LAB co-fermentation represents a feasible strategy for the valorization of PNC into functional peptide ingredients. Further studies are needed to validate these effects in cellular and animal models, particularly with respect to the Keap1-Nrf2 signaling pathway.

## Figures and Tables

**Table 1:** Amino acid content.

Name	Retention Time [min]	Area [mV.s]	Content [mg/g]
Aspartic acid	8.379	356.264	0.932
Threonine	10.185	307.785	0.747
Serine	11.211	210.056	0.39
Glutamic acid	13.429	591.654	1.41
Glycine	19.652	196.309	0.231
Alanine	20.94	292.439	0.418
Cysteine	22.583	132.629	0.713
Valine	23.203	106.165	0.23
Methionine	24.993	316.762	0.676
Isoleucine	26.693	221.234	0.588
Leucine	27.42	129.101	0.31
Tyrosine	29.841	82.201	0.286
Phenylalanine	30.716	90.616	0.263
Histidine	35.505	83.632	0.233
Lysine	36.845	139.955	0.373
Arginine	45.029	111.59	0.316
Total		3368.392	8.117

**Table 2:** Comprehensive Molecular Docking Results of the Two Peptide Segments with SOD, Keap1, and α-Glucosidase.

Receptor Protein	SOD				Keap1				α-Glucosidase			
Polypeptide	FAGDDAPR		LAGNPDDEFRPQ		FAGDDAPR		LAGNPDDEFRPQ		FAGDDAPR		LAGNPDDEFRPQ	
Molecular docking binding energy (kcal/mol)	−5.8		−5.6		−9.8		−9.4		−7.9		−5.8	
Mode of action	Hydrogen bond	Hydrophobic interaction	Hydrogen bond	Hydrophobic interaction	Hydrogen bond	Hydrophobic interaction	Hydrogen bond	Hydrophobic interaction	Hydrogen bond	Hydrophobic interaction	Hydrogen bond	Hydrophobic interaction
Hydrogen-bonding residues and hydrophobic residues of the receptor protein	Ser-3, His-71, Ser-75, Gln-147, Arg-192	Pro-5, Ile-76, Glu-191, Ala-195	Ser-3, His-71, Ser-75, Gln-147, Arg-192, Ala-195	Leu-38, Ile-72, Ile-76, Pro-154	Ser-363, Asn-414, Arg-415, Arg-483, Ser-508, Gln-530, Ser-555, Leu-557	Tyr-334, Ala-366, Tyr-535	Tyr-334, Asn-414, Arg-415, Ile-416, His-436, Ser-555, Tyr-572, Val-604	Tyr-334, Tyr-525, Phe-577	Gly-228, Glu-231, Leu-300, Asn-301, Glu-396	Phe-147, Thr-203, Ala-224, Leu-227, Met-302, Tyr-389, Phe-397	Phe-62, Thr-226, Gly-228, Glu-231, Asp-274, Asp-275, Asp-333, Glu-377, Tyr-389, Ile-377, Glu-396	Phe-147, Phe-166, Lys-225, Tyr-235, Pro-277, Phe-297, Asn-301, Pro-303, Tyr-308, Val-334, Val-335, Phe-397

## Data Availability

The original contributions presented in this study are included in the article/Supplementary Materials. Further inquiries can be directed to the corresponding author.

## Supplementary Materials

The following supporting information can be downloaded at: https://www.mdpi.com/article/10.3390/foods15020323/s1. Figure S1. Effects of PNCH on body weight and liver index in mice; Figure S2. Basic information analysis of peptides derived from PNCH. (A) Total ion chromatogram (TIC) of peptides; (B) peptide length distribution; (C) peptide molecular weight distribution; (D) C-terminal amino acid composition; (E) N-terminal amino acid composition; (F) overall amino acid composition of peptides; Figure S3. Purity analysis of peptides (A) FAGDDAPR and (B) LAGNPDDEFRPQ; MS spectra of peptides (C) FAGDDAPR and (D) LAGNPDDEFRPQ; Figure S4. The effects of (A) peptides at different concentrations and (B) H_2_O_2_ on cell viability; Table S1. Results of in silico screening and characterization of different peptides.
