# Supplementary material for: Unlocking the Functional Potential of Pecan Nut Cake: A Study on Bioactive Peptide Production
Source: Foods. 2026 Jan 15;15(2):323. doi: 10.3390/foods15020323 (PMC12841524; doi:10.3390/foods15020323)
Supplement: Supplementary file 1 [file foods-15-00323-s001.zip › foods-4077939-supplementary.pdf]

Supporting Information for

Unlocking the Functional Potential of Pecan Nut Cake: A Study on Bioactive Peptide Production

Tianjing Long[1#], Yingjie Xu[1,2#], Ziang Li[1], Weimei Kong[1], Yibo Zhu[2], Mingxun Tao[1], Haibo Luo[1], Li Cui[3], Mingjun Sun[4], Zhen Wu[5], Xiaoqun Zeng[5], Daodong Pan[5], Yuxing Guo[1*]

1 Department of Food Science and Technology, School of Food Science and Pharmaceutical Engineering, Nanjing Normal University, Nanjing 210097, China
2 School of Biological and Food Engineering, Suzhou Institute of Technology, Suzhou 215500, China
3 Institute of Agro-Product Processing, Jiangsu Academy of Agricultural Sciences, Nanjing 210014, China
4 Nanjing You Neng Biotechnology Co., Ltd., Nanjing 211200, China
5 School of Food Science and Engineering, Ningbo University, Ningbo 315800, China
* Correspondence: **Yuxing Guo**-Department of Food Science and Technology, School of Food Science and Pharmaceutical Engineering, Nanjing Normal University, Nanjing 210097, China; orcid.org/0000-0003-1285-2545; Tel.:+86-(0)25-85898184; fax:+86-(0)25–858981846, Nanjing 210097, E-mail: guoyuxing1981@163.com.
# Tianjing Long and Yingjie Xu contributed equally to this manuscript.

The Supplementary materials includes:

Figure S1 Effects of PNCH on body weight and liver index in mice;

Figure S2 Basic information analysis of peptides derived from PNCH. (A) Total ion chromatogram (TIC) of peptides; (B) peptide length distribution; (C) peptide molecular weight distribution; (D) C-terminal amino acid composition; (E) N-terminal amino acid composition; (F) overall amino acid composition of peptides;

Figure S3 Purity analysis of peptides (A) FAGDDAPR and (B) LAGNPDDEFRPQ; MS spectra of peptides (C) FAGDDAPR and (D) LAGNPDDEFRPQ;

Figure S4 The effects of (A) peptides at different concentrations and (B) H2O2 on cell viability.

Table S1 Results of in silico screening and characterization of different peptides.

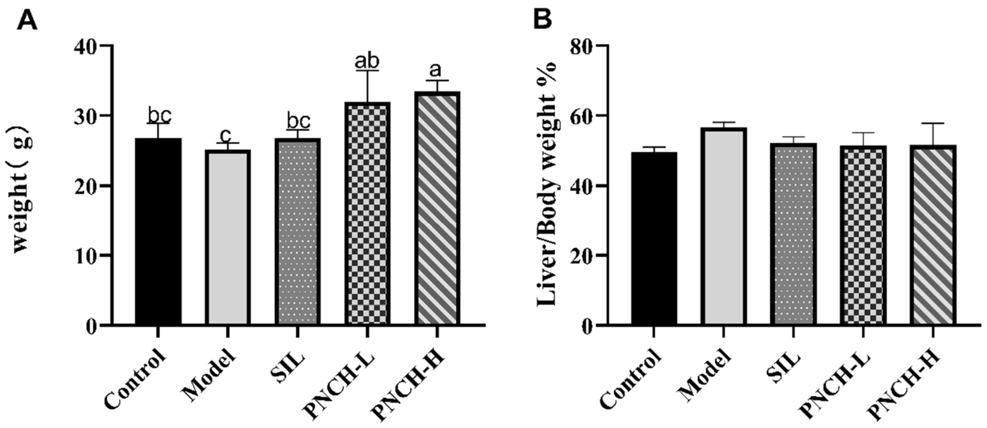

Figure S1 Effects of PNCH on body weight and liver index in mice

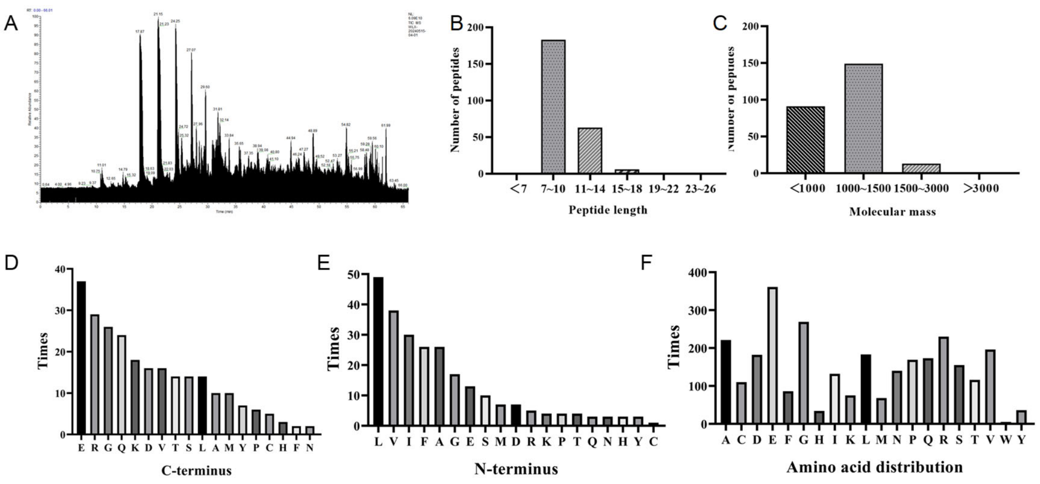

Figure S2 Basic information analysis of peptides derived from PNCH. (A) Total ion chromatogram (TIC) of peptides; (B) peptide length distribution; (C) peptide molecular weight distribution; (D) C-terminal amino acid composition; (E) N-terminal amino acid composition; (F) overall amino acid composition of peptides

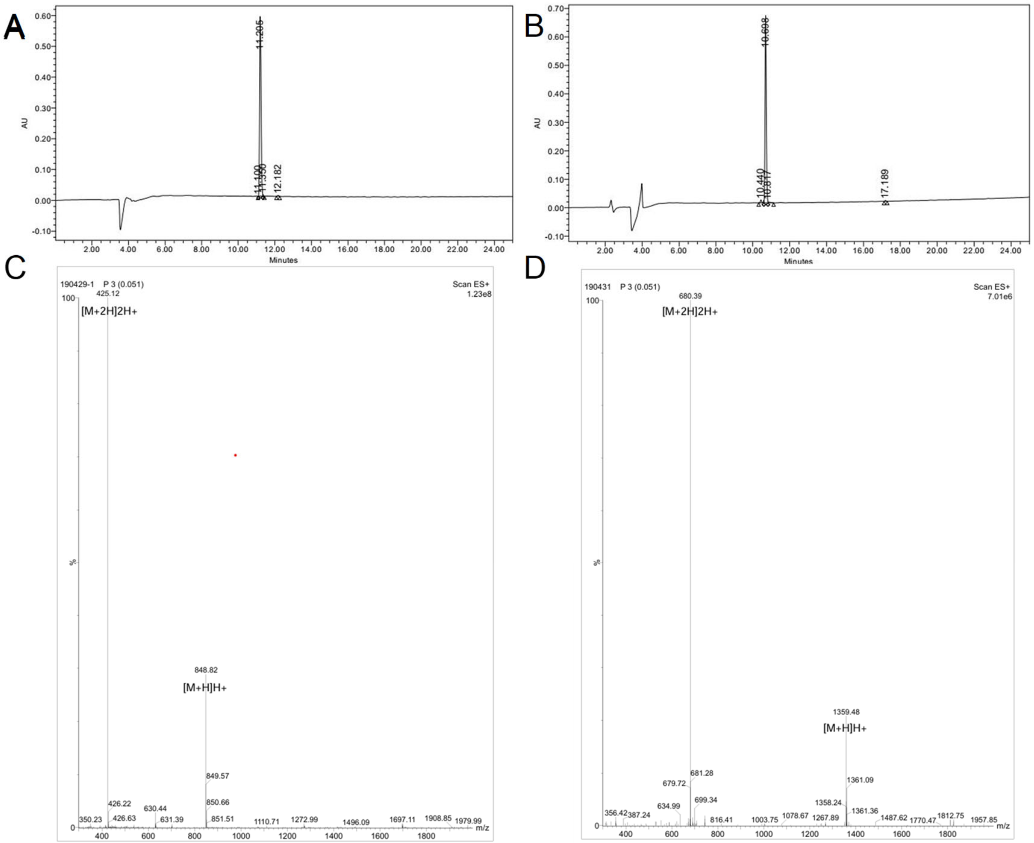

Figure S3 Purity analysis of peptides (A) FAGDDAPR and (B) LAGNPDDEFRPQ; MS spectra of peptides (C) FAGDDAPR and (D) LAGNPDDEFRPQ.

As shown in Figure S4, the cytotoxicity of peptides was evaluated using the MTT assay in Caco-2 cells. Both FAGDDAPR and LAGNPDDEFRPQ exhibited good cellular tolerance at concentrations up to 400 μg/mL, with cell viability remaining above approximately 85% relative to the control group. In contrast, a more pronounced decline in cell viability was observed at 800 μg/mL, indicating potential concentration-dependent stress at higher peptide levels. Therefore, 400 μg/mL was selected as an appropriate peptide concentration for subsequent cellular experiments, balancing cellular safety and experimental relevance. To establish a stable oxidative stress model, Caco-2 cells were exposed to increasing concentrations of H_2O_2 (25–600 μM). Cell viability decreased in a dose-dependent manner, with 200 μM H_2O_2 reducing viability to approximately 45-55%, closely matching the commonly accepted target range for oxidative injury models. Higher concentrations (⩾ 400 μM) resulted in excessive cytotoxicity, which could interfere with the evaluation of protective effects. Accordingly, 200 μM H_2O_2 was selected as the optimal concentration for inducing oxidative stress in subsequent experiments.

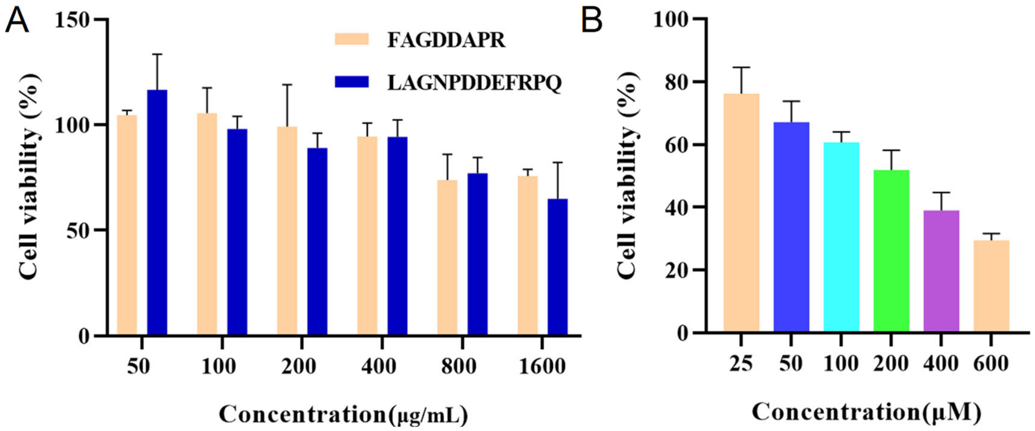

Figure S4 The effects of (A) peptides at different concentrations and (B) H_2O_2 on cell viability.

Table S1 Results of in silico screening and characterization of different peptides

Sequences	SCCGGNCGCGTGCKCGSDCGGCKE	GCKCGSSCSCDPCNCK	FAGDDAPR	LAGNPDDEFRPQ
Molecular mass	2685.852	1977.657	847.3824	1357.626
Lengths	24	16	8	12
Peptide ranker score	0.999831	0.859745	0.641361	0.566446
Toxin	Non	Non	Non	Non
allergen	Non	Non	Non	Non
TheoreticalpI	5.77	7.77	4.21	4.03
previously reported	Not	Not	Yes	Not
Intestinal stability	High	High	High	High
Half-Life in plasma (s)	829.71	816.61	845.51	820.71
